# Molecular insights into the surface-catalyzed secondary nucleation of amyloid-β_40_ (Aβ_40_) by the peptide fragment Aβ_16–22_

**DOI:** 10.1126/sciadv.aav8216

**Published:** 2019-06-21

**Authors:** Samuel J. Bunce, Yiming Wang, Katie L. Stewart, Alison E. Ashcroft, Sheena E. Radford, Carol K. Hall, Andrew J. Wilson

**Affiliations:** 1 School of Chemistry, University of Leeds, Leeds LS2 9JT, UK.; 2 Astbury Centre for Structural Molecular Biology, University of Leeds, Leeds LS2 9JT, UK.; 3 Department of Chemical and Biomolecular Engineering, North Carolina State University, Raleigh, NC 27695-7905, USA.; 4 School of Molecular and Cellular Biology, Faculty of Biological Sciences, University of Leeds, Leeds LS2 9JT, UK.

## Abstract

Understanding the structural mechanism by which proteins and peptides aggregate is crucial, given the role of fibrillar aggregates in debilitating amyloid diseases and bioinspired materials. Yet, this is a major challenge as the assembly involves multiple heterogeneous and transient intermediates. Here, we analyze the co-aggregation of Aβ_40_ and Aβ_16–22_, two widely studied peptide fragments of Aβ_42_ implicated in Alzheimer’s disease. We demonstrate that Aβ_16–22_ increases the aggregation rate of Aβ_40_ through a surface-catalyzed secondary nucleation mechanism. Discontinuous molecular dynamics simulations allowed aggregation to be tracked from the initial random coil monomer to the catalysis of nucleation on the fibril surface. Together, the results provide insight into how dynamic interactions between Aβ_40_ monomers/oligomers on the surface of preformed Aβ_16–22_ fibrils nucleate Aβ_40_ amyloid assembly. This new understanding may facilitate development of surfaces designed to enhance or suppress secondary nucleation and hence to control the rates and products of fibril assembly.

## Teaser

Combined experiment and simulation reveals a structural mechanism of surface-catalyzed nucleation in Aβ amyloid formation.

## INTRODUCTION

Understanding the molecular mechanisms of peptide self-assembly into amyloid fibrils is of key importance in understanding pathological disease states (*1*) and in designing new functional materials (*2*). Aberrant self-assembly of monomeric peptides or proteins into amyloid fibrils is associated with a number of degenerative conditions, notably, Alzheimer’s and Parkinson’s disease (*1*, *3*), in which considerable evidence now implicates soluble oligomers as the primary cause of cellular damage (*4*, *5*). Identifying and characterizing the structural changes that occur during peptide assembly into amyloid fibrils is essential in the quest to develop strategies to combat disease and manufacture bespoke materials (*1*, *6*).

Peptide assembly into amyloid fibrils occurs via a complex nucleation-dependent mechanism, in which subtle changes in lowly populated states can have marked effects on the rates and products of assembly (*7*). Elegant work has resulted in kinetic models that are able to dissect the different contributing steps in assembly, including primary nucleation, elongation, fragmentation, and secondary nucleation (*8*–*11*). Secondary nucleation is the process whereby transient binding to a fibril surface accelerates aggregation by promoting the formation of nuclei on the fibril surface. The activation energy barrier for this phase of aggregation for Aβ_42_ has been shown to be enthalpic (*11*) and distinct from that of other kinetic phases of assembly. Secondary nucleation is thought to be a specific process in which the effectiveness of nucleation can depend on the sequence and morphology of both the fibril and the assembling monomers, although the “rules” defining this specificity have yet to be elucidated. However, elucidating structural insights into these different steps in assembly, including the nature of early oligomeric species, is challenging, as circular dichroism (CD), infrared, and other spectroscopic techniques generally only observe population-average data for a whole system. Single-molecule Förster resonance energy transfer (FRET) and solid-state nuclear magnetic resonance (NMR), which have uncovered clues as to the structure of toxic versus nontoxic oligomeric species (*12*, *13*), provide information on the average properties of the different species at different times. Native ion mobility spectrometry–mass spectrometry (IMS-MS) separates ions based on shape as well as mass and charge (*14*) and has been used to provide insights into the population, conformation, and ligand-binding capability of individual peptide monomers and oligomers (*15*). By using photo-induced cross-linking (PIC), fleeting interpeptide/intrapeptide interactions may be trapped through covalent bond formation (to encode supramolecular connectivity) (*16*). Molecular dynamics (MD) simulations focusing on multipeptide systems at short time scales (<1 ms) (*17*) can help fill the gaps between population-average data and individual structures. Such simulations can provide insights into self-assembly events in molecular detail, allowing the earliest stages of aggregation to be visualized and the course of aggregation to be tracked in all-atom detail (*18*–*20*).

**Fig. 1:**
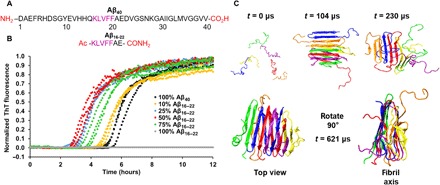
Co-aggregation of Aβ_16–22_ and Aβ_40_ results in accelerated aggregation kinetics for Aβ_40_. (**A**) Primary sequence of Aβ_16–22_ and Aβ_40_, including the groups at each terminus. The central recognition motif KLVFF is highlighted in purple. (**B**) ThT fluorescence assays showing that the aggregation rate of Aβ_40_ increases as the ratio of Aβ_16–22_ to Aβ_40_ is increased (with the total peptide concentration held constant at 40 μM). (**C**) Simulation snapshots of the aggregation of six Aβ_40_ monomers into a β-sheet–rich hexamer at an Aβ_40_ concentration of 5 mM. At the start of the simulation (0 μs), all the peptides are in random coils, but as the simulation progresses, they aggregate into antiparallel, in-register β sheets (104 μs). This oligomer then unfolds, losing some of its β-sheet structure (230 μs) before a rearrangement in which the β sheets rearrange, forming a stable fibril, with each Aβ_40_ peptide containing three β strands (621 μs) engaged in parallel intermolecular hydrogen bonding.

The amyloid-β peptide (Aβ) is a major component of the extracellular plaques observed in Alzheimer’s disease (*5*, *21*). Aggregation of Aβ_40/42_ (Fig. 1A) into amyloid fibrils has been widely studied both in vitro and in vivo (*22*), although numerous questions remain about its structure and role in Alzheimer’s disease progression (*1*, *22*). Kinetic analysis of the sigmoid growth curves of Aβ_40/42_ aggregation has enabled their assembly mechanisms to be deconvoluted into a number of microscopic steps (*7*, *10*). Assembly begins with a lag phase, during which time monomers and small amounts of oligomers persist (*7*). Monomers then undergo a rearrangement step to form a nucleus (primary nucleation) from which fibrils can grow. Further aggregate growth occurs through pathways that include elongation (whereby a monomeric peptide adds onto the end of a growing fibril), fragmentation (fibrils break into two smaller aggregates, exponentially increasing growth-competent fibril ends), and surface-catalyzed secondary nucleation (whereby nucleation is catalyzed on the fibril surface) (*23*). Using MD simulations, the energy landscape of Aβ_40_ oligomer formation has also been modeled, demonstrating the different kinetic pathways that underlie the formation of prefibrillar and nonfibrillar oligomers (*17*). For Aβ_40_, primary nucleation has been shown to be a slower process than secondary pathways such that surface-catalyzed secondary nucleation events dominate the growth rate of fibrils (*10*). Under quiescent conditions, the contribution of fibril fragmentation to the growth of fibrils has been shown to be negligible (*9*). Co-aggregation processes (i.e., where two different peptide sequences interact during aggregation but need not co-assemble) can result in more complex kinetics due to the possibility of the sequences interacting with each other to modulate aggregation (*24*, *25*). Such a situation may occur in vivo wherein multiple sequences of different lengths of Aβ are formed (*26*).

Here, we combine fluorescence assays, electrospray ionization (ESI)–IMS–MS, and PIC experiments to study the structural mechanism of co-assembly of the peptide fragment Aβ_16–22_ (Fig. 1A), which contains the “core recognition motif” KLVFF (*27*) of Aβ_40_, with the parent Aβ_40_ sequence. Aβ_16–22_ has been shown to form fibrils with an in-register, antiparallel orientation at neutral pH (*28*) and has been proposed to assemble via an intermediate with out-of-register β-sheet alignment before reaching the final in-register fibril structure (*29*). The rate of Aβ_16–22_ aggregation is dependent on peptide concentration and ionic strength (*29*–*31*). Discontinuous MD (DMD) has also shown that the nucleation-dependent aggregation process of Aβ_16–22_ proceeds from a random coil configuration to form multilayer β-sheet fibrils, with an in-register antiparallel β-sheet orientation, in accordance with the experimental data (*32*). Here we show, using fluorescence quenching assays, that Aβ_16–22_ aggregates more rapidly than Aβ_40_ and that Aβ_16–22_ fibril formation then increases the aggregation rate of Aβ_40_ through a surface-catalyzed secondary nucleation mechanism, mirroring the behavior observed in kinetic analyses of Aβ_40/42_ aggregation (*9*, *10*) and their co-aggregation (*24*). Using DMD simulations, we also show that the preformed Aβ_16–22_ fibrils increase the early-stage aggregation rate of Aβ_40_ but that the monomeric Aβ_16–22_ peptides do not, supporting secondary nucleation as the mechanism of enhanced Aβ_40_ aggregation by Aβ_16–22_. These experimentally validated simulations portray the structural mechanism of surface-catalyzed nucleation. This new understanding may pave the way to the generation of surfaces able to enhance or suppress assembly and may inform effective design of ligands that modulate therapeutically important amyloid assembly.

## RESULTS

### Aβ_16–22_ increases the aggregation rate of Aβ_40_

**Fig. 2:**
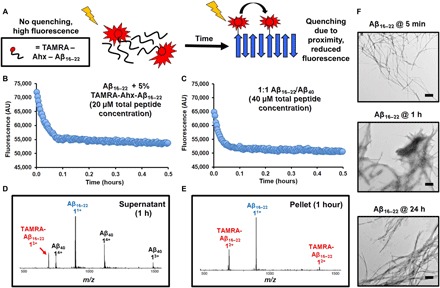
Aggregation kinetics of Aβ_16–22_ are unaffected by the presence of Aβ_40_. (**A**) Schematic showing the principle behind the fluorescence quenching assay used to determine the aggregation rate of Aβ_16–22_. (**B**) As self-assembly occurs, the TAMRA-labeled peptides [40 μM total peptide containing 5% (w/w) TAMRA-Ahx-Aβ_16–22_] are sequestered into the fibril structure. This brings the fluorophores into proximity, resulting in fluorescence quenching. (**C**) Aggregation of Aβ_16–22_ [containing 5% (w/w) TAMRA-Ahx-Aβ_16–22_] and Aβ_40_ at a 1:1 mol/mol ratio (40 μM total peptide concentration). A single transient, which is the median of three replicates measured, is shown. (**D** and **E**) Sedimentation and separation of the pellet and supernatant of the 1:1 mixed system and analysis of the fractions using ESI-MS after 1 hour indicate that Aβ_40_ is present in the (D) supernatant and in only very small amounts within the (E) pellet. (**F**) Under these conditions, fibrils of Aβ_16–22_ are present after 5 min of incubation. Scale bars, 500 nm.

To determine whether the presence of Aβ_16–22_ affects the aggregation rate of Aβ_40_, we synthesized or recombinantly expressed the peptides (see Materials and Methods, Supplementary Materials, and figs. S1 and S2), purified them, and mixed them in different ratios at a constant total peptide concentration of 40 μM. The rate of aggregation was then measured using the fluorescence of thioflavin-T (ThT; Fig. 1B, Materials and Methods, and fig. S3). Initial experiments showed the expected sigmoid increase in ThT fluorescence for Aβ_40_ (*7*, *10*, *33*), indicating the assembly of this peptide into amyloid fibrils (Fig. 1B). While Aβ_16–22_ formed fibrils under the conditions used based on transmission electron microscopy (TEM) images (Fig. 2F and fig. S4), as expected (*16*), ThT fluorescence did not increase over 12 hours (Fig. 1B), indicating that the fibrils formed either are unable to bind ThT or do not enhance its fluorescence when bound; rotational immobilization of ThT is required for its fluorescent enhancement when bound to amyloid fibrils (*34*). Other amyloid dyes (NIAD-4, Congo Red, and ANS) were screened against Aβ_16–22_ fibrils; however, none produced a signal with which to perform kinetic assays. The increase in ThT signal in the peptide mixture thus reports on the aggregation rate of Aβ_40_ and how this is affected by the presence of Aβ_16–22_. The experiments in Fig. 1B show that, at a constant peptide concentration of 40 μM, as the molar ratio of Aβ_16–22_ to Aβ_40_ is increased, the apparent aggregation rate of Aβ_40_ also increases. Competition between the increased rate of Aβ_40_ aggregation as Aβ_16–22_ concentration increases and the decreased rate of aggregation of Aβ_40_ as its concentration correspondingly decreases results in maximal apparent rate enhancement at a 1:1 molar ratio of the two peptides (Fig. 1B). We accounted for this effect by measuring, in parallel, the *t*_1/2_ (the time at which the growth curve reaches 50% amplitude) value of aggregation of Aβ_40_ alone at each concentration and comparing the *t*_1/2_ values with and without Aβ_16–22_ added (see fig. S3). These data show that the effect saturates as would be expected for secondary nucleation events involving binding to the fibril surface.

To characterize the extent to which Aβ_40_ aggregation is accelerated by the presence of Aβ_16–22_, we calculated the half-time (*t*_1/2_) for each peptide mixture and normalized to the half time for the equivalent concentration of Aβ_40_ alone (fig. S3). The results revealed a marked, and titratable, effect of the presence of Aβ_16–22_ on the aggregation rate of Aβ_40_, demonstrating an interaction between the two peptides that accelerates the rate of assembly.

### Aβ_16–22_ aggregates more rapidly than Aβ_40_ and is unaffected by the presence of Aβ_40_

As the assembly kinetics of Aβ_16–22_ could not be measured using any of the amyloid dyes surveyed at the concentrations used here, a fluorescence quenching assay was developed to determine whether Aβ_16–22_ aggregates more or less rapidly than Aβ_40_ (Fig. 2A). Similar assays have been previously used to monitor the aggregation rates of Aβ_40_ and Aβ_42_ (*35*), with fluorescence quenching reporting on labeled monomers coming into mutual proximity as oligomers (or fibrils) form. For these assays, Aβ_16–22_ N-terminally labeled with tetramethylrhodamine (TAMRA) was synthesized, including a 6-aminohexanoic acid linker (Ahx) to limit disruption to the native fibril structure that might arise due to the bulky fluorophore (TAMRA-Ahx-Aβ_16–22_; Supplementary Materials and figs. S1 and S4). When incubated in isolation, a 5% (w/w) TAMRA-Ahx-Aβ_16–22_:95% Aβ_16–22_ mixture (20 μM) resulted in a rapid decrease in fluorescence intensity followed by a slower phase that plateaued after 1 hour (Fig. 2B). In the presence of Aβ_40_ [1:1 (mol/mol) ratio, 40 μM total peptide concentration, and 2% (v/v) dimethyl sulfoxide (DMSO)], no difference in the rate of fluorescence decrease was observed, indicating that the presence of Aβ_40_ has no effect on Aβ_16–22_ aggregation (Fig. 2C). Analysis of these samples by negative-stain TEM showed the presence of fibrils after only 5 min (Fig. 2F). Sedimentation of the mixed system by centrifugation after 1 hour demonstrated that Aβ_40_ was present mainly in the supernatant (Fig. 2, D and E). These results demonstrate that Aβ_16–22_ aggregates rapidly to form amyloid-like fibrils, while Aβ_40_ remains soluble as monomers/oligomers. Thus, although the rate of Aβ_40_ aggregation is increased by the presence of Aβ_16–22_, limited or no co-assembly between the two peptides into fibrils was observed. By contrast, Aβ_16–22_ aggregation is unaffected by the presence of Aβ_40_. Aβ_40_ fibrils have been shown to adopt a parallel in-register structure involving most of the polypeptide backbone (*21*, *36*), while Aβ_16–22_ has been shown to form an antiparallel β-stranded amyloid structure (*28*, *29*). This structural incompatibility could account for the absence of co-assembly because such a structure would be less stable compared with homomeric assemblies. Furthermore, the more rapid fibril assembly of Aβ_16–22_ in comparison to Aβ_40_ disfavors co-assembly on kinetic grounds.

### Monomeric Aβ_16–22_ can interact with monomeric and oligomeric Aβ_40_ through the self-recognition motif KLVFF

**Fig. 3:**
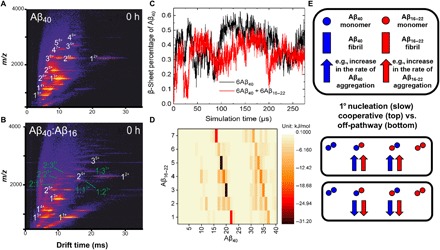
Aβ_16–22_ can interact with Aβ_40_ monomers and dimers. (**A**) Native ESI-IMS-MS drift-scope images of Aβ_40_ indicate the presence of multiple oligomeric species of Aβ_40_ (white numbers). (**B**) When mixed at a 1:1 mol/mol ratio with Aβ_16–22_ (yellow numbers), a number of heteromeric species are observed (light blue numbers) immediately following mixing. The oligomer size is given (1, 2, 3, etc.), with the charge state in superscript. (**C**) DMD simulation showing the percent β sheet formed by Aβ_40_ during aggregation in the absence (black) or presence (red) of Aβ_16–22_. (**D**) Energy contact map between one monomer of Aβ_16–22_ and one of Aβ_40_ scaled by energy (bar shown alongside), showing that residues 17 to 20 (LVFF) and 31 to 34 (IIGL) form the strongest interactions. (**E**) Co-aggregation can have differing effects on the primary nucleation of each peptide, depending on whether the mixed oligomers formed can progress to form mixed fibrils or are off-pathway and take no further part in the aggregation reaction. Circles represent monomers and blocks represent fibrils, with Aβ_16–22_ and Aβ_40_ in red and blue, respectively. Adapted from (*24*).

To determine whether Aβ_16–22_ and Aβ_40_ interact transiently in the early stages of assembly, we performed native ESI linked to IMS-MS (see Materials and Methods). This soft ionization technique has been used to identify and structurally characterize amyloid oligomers that formed from several different proteins and peptides (*14*, *15*). Under the conditions used here, ESI-IMS-MS immediately following mixing revealed that Aβ_40_ copopulates a number of oligomers, ranging from monomers to pentamers (Fig. 3B, white, and table S1), consistent with previous results (*33*). When incubated with Aβ_16–22_, heteromolecular oligomers were observed (Fig. 3B, light blue), along with homomolecular oligomers of Aβ_40_ (Fig. 3A, white). Notably, Aβ_16–22_ homomolecular oligomers were not observed. The heteromolecular oligomers correspond to multiple Aβ_16–22_ monomers that bound to either an Aβ_40_ monomer or dimer (table S1). Collision cross-section (CCS) estimations from the ESI-IMS-MS analysis of the Aβ_40_ species in the presence or absence of Aβ_16–22_ indicate no discernible difference in the gas-phase cross-section of Aβ_40_, implying that a conformational change in monomer or oligomer structure is unlikely to be the provenance for the Aβ_16–22_-driven increase in the Aβ_40_ aggregation rate (fig. S5). Despite attempts to capture the interaction experimentally by PIC using a diazirine-labeled Aβ_16–22_ (Aβ*_16–22_; see the Supplementary Materials for synthesis, scheme S1, and fig. S1), the site of interaction could not be verified (fig. S6 and table S2) likely due to the low percentage of any heterodimers present (as assessed by total ion count, 1.0 ± 0.5%) and the lower solution concentration of Aβ_16–22_ arising as a consequence of its rapid aggregation.

To further assess the nature of the interactions between Aβ_16–22_ and Aβ_40_, we performed DMD simulations (see Materials and Methods). To evaluate the role of interactions between Aβ_40_ and Aβ_16–22_ monomers (covered in this section), it was first necessary to perform DMD simulations on the aggregation of Aβ_40_ alone (Fig. 1C) and then a 1:1 mixture of the Aβ_16–22_ and Aβ_40_ peptide sequences at *C*_peptide_ = 5 mM (Fig. 3C). These co-aggregation simulations starting from monomeric peptides are further discussed in the course of our analyses to rule out co-assembly (see below), and then we describe DMD analyses on the effect of Aβ_16–22_ fibrils on Aβ_40_ aggregation (see below). Simulations performed on six monomers of Aβ_40_ (Fig. 1C) showed that the initially unstructured peptides assemble and adopt a metastable oligomer structure by 104 μs (Fig. 1C); this structure comprises antiparallel intramolecular β strands linked by disordered regions assembled into antiparallel intermolecular sheets, with β strands stacked perpendicular to the long axis. During this oligomerization stage, the peptide conformation is similar to that observed by Zheng *et al.* (*17*). As the simulation proceeds, this oligomer loses some β-sheet content (*t* = 230 μs; Fig. 1C). By the end of the simulation (621 μs), peptides in oligomers undergo structural rearrangement from antiparallel β-strand conformations to the parallel β-sheet conformation observed for Aβ_40_ fibrils (Fig. 1C) (*37*). Simulations of the peptide mixtures did not show an accelerating effect of Aβ_16–22_ monomers on the aggregation rate of Aβ_40_ (see Fig. 3C and later). However, interactions between the two peptides were observed, consistent with the ESI-MS results in Fig. 3. From the DMD data, an energy contact map between the monomeric Aβ_16–22_ and Aβ_40_ peptides was calculated (Fig. 3D). The contact map indicated that Aβ_16–22_, specifically residues 18 to 20 (VFF), interacts strongly with residues 19 to 21 and 32 to 35 of Aβ_40_ (FFA and IGLM, respectively), consistent with experimental data previously reported, which indicate that KLVFF is a “self-recognition element” (*27*). Such an interaction between Aβ_16–22_ and Aβ_40_ oligomers, however, does not result in an acceleration of aggregation (Fig. 3C), implying that these mixed and low-abundance oligomers represent transient species that do not affect the rate of assembly (Fig. 3E).

### Aβ_16–22_ fibrils have a larger effect on the aggregation rate of Aβ_40_ than Aβ_16–22_ monomer

**Fig. 4:**
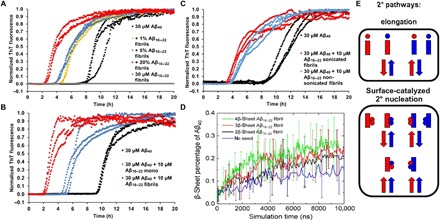
Aβ_16–22_ fibrils increase the aggregation rate of Aβ_40_ to a greater extent than Aβ_16–22_ monomers. (**A**) Increased concentrations (% w/w) of Aβ_16–22_ fibrils were added to Aβ_40_ monomers (as shown in the key), and the aggregation rate was measured by ThT fluorescence. (**B**) Direct comparison of the effect of Aβ_16–22_ monomers (i.e., taken straight from a DMSO stock) and Aβ_16–22_ fibrils on Aβ_40_ aggregation. (**C**) Effect of sonicating the Aβ_16–22_ fibrils on the Aβ_40_ aggregation rate shows little effect compared with the data shown in (A) (see text for details). (**D**) Plots of the percent β sheet formed by Aβ_40_ in the absence (blue) or presence of preformed two (black), three (red), or four (green) β-sheet Aβ_16–22_, determined using DMD, showing that an increased Aβ_16–22_ fibril size increases the rate of Aβ_40_ aggregation. (**E**) During co-aggregation experiments, both elongation and surface-catalyzed mechanisms can occur; each has a different effect on the rate of assembly of each peptide (the same notation is used as in Fig. 3E, with circles representing monomers, blocks representing fibrils, and Aβ_16–22_ and Aβ_40_ in red and blue, respectively). Adapted from (*24*).

To determine whether rapidly formed Aβ_16–22_ fibrils are the causative agents of the enhanced rate of Aβ_40_ aggregation in the mixed samples (Fig. 1B), we assessed the effect of preformed Aβ_16–22_ fibrils on Aβ_40_ aggregation. These experiments (Fig. 4A) showed that the presence of Aβ_16–22_ fibrils increases the rate of aggregation of Aβ_40_ in a fibril concentration–dependent manner (Fig. 4A), and addition of Aβ_16–22_ fibrils had a larger effect on the aggregation rate compared with the addition of monomeric (i.e., taken straight from a DMSO stock) Aβ_16–22_ (Fig. 4B). This suggests that aggregation is enhanced either by cross-seeding (i.e., by adding Aβ_40_ directly to the ends of Aβ_16–22_ fibrils) or by secondary nucleation of Aβ_40_ on the Aβ_16–22_ fibril surface (Fig. 4E). Sonication of fibrils fragments them, leading to a higher concentration of fibril ends. Hence, should elongation dominate the rate of fibril formation, sonication should markedly increase the rate of fibril growth. Comparison of the effects of unsonicated fibrils (fewer ends) with the same fibrils fragmented by sonication (Fig. 4C and see fig. S4 for TEM analyses) indicated that elongation was not dominant (Fig. 4C), because the average *t*_1/2_ for sonicated fibrils (6.2 ± 1.0 hour) is similar to that of its unfragmented counterpart (7.2 ± 0.7 hours). Together, the results demonstrate that the presence of rapidly formed Aβ_16–22_ fibrils enhances aggregation of Aβ_40_ in peptide mixtures by secondary nucleation, despite the presence of small amounts of mixed oligomers (as demonstrated by the ESI-IMS-MS experiments).

DMD simulations of the aggregation of six Aβ_40_ peptides were also performed in the presence of preformed Aβ_16–22_ fibrils of different sizes (two, three, and four β sheets) at an Aβ_40_ concentration of 1 mM to model the dynamic process of the secondary nucleation event. The results (Fig. 4D) showed that the largest Aβ_16–22_ fibril (i.e., four β sheets; green trace in Fig. 4D) led to the largest increase in the rate of β-sheet formation by Aβ_40_. Given that the presence of Aβ_16–22_ monomers had no observable effect on Aβ_40_ assembly (Fig. 3C), these simulations are thus qualitatively concordant with the experimental findings that the fibrillar structure of Aβ_16–22_ is the dominant influence on the aggregation rate of Aβ_40_. Such behavior is consistent with that observed for Aβ_40/42_ co-aggregation for which a kinetic model has been established (*24*).

### Aβ_40_ and Aβ_16–22_ form distinct homomolecular fibrils

The peptide composition of the final fibril structure(s) represents a further means to discern the difference between surface-catalyzed secondary nucleation and co-assembly exploiting fibril ends. A surface-catalyzed mechanism would most likely produce homomolecular fibrils of Aβ_40_, as once they have formed on the Aβ_16–22_ fibril surface, the Aβ_40_ nuclei would dissociate and form pure Aβ_40_ fibrils. In contrast, co-assembly involving fibril ends should result in mixed fibrils, in which Aβ_16–22_ seeds are segmentally separated from fibril regions containing Aβ_40_ monomers.

**Fig. 5:**
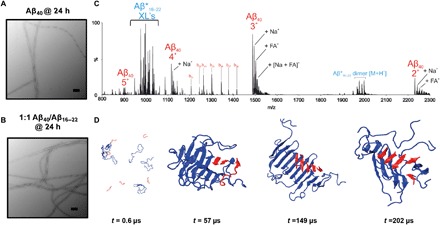
Aβ_16–22_and Aβ_40_do not co-assemble during co-aggregation. Negative-stain TEM analysis of Aβ_40_ incubated for 24 hours in the (**A**) absence or (**B**) presence of Aβ_16–22_. Scale bars, 200 nm. (**C**) PIC of mixtures of diazirine-labeled Aβ_16–22_ (Aβ*_16–22_) and Aβ_40_ incubated for 24 hours and then irradiated for 30 s. Only homomolecular Aβ_16–22_ cross-links are observed, indicating that the fibrils are not copolymerized at the end of the reaction (the inset depicts the mechanism of PIC of the diazirine group. (**D**) DMD simulation snapshots of co-aggregation of Aβ_40_ (blue) and Aβ_16–22_ (red) indicate that separate homomolecular oligomers are formed at *t* = 202 μs.

Negative-stain TEM images taken at the end of the aggregation reaction showed Aβ_40_ fibrils with similar gross morphology when incubated in isolation or co-aggregated with Aβ_16–22_ (Fig. 5, A and B). Similarly, quantitation of ThT fluorescence at the end-point of aggregation in mixed samples and quantitation of the same concentration of Aβ_40_ incubated alone were indistinguishable (fig. S3), supporting the hypothesis that homomolecular Aβ_40_ fibrils are formed at the end of the assembly reaction. Last, PIC was used to explore whether homo- or heteromolecular fibrils had formed (Fig. 5C and Materials and Methods). To perform PIC experiments, a diazirine label was placed on F20 of Aβ_16–22_ (Aβ*_16–22_) (*16*). Control experiments demonstrated that Aβ*_16–22_ has a similar effect on the rate of Aβ_40_ aggregation as its unmodified counterpart (fig. S3). PIC experiments performed 5 min and 24 hours after initiating assembly failed to detect cross-links between Aβ*_16–22_ and Aβ_40_ (Fig. 5C, fig. S6, and table S2). Instead, all identifiable cross-links were consistent with intermolecular/intramolecular Aβ*_16–22_ or solvent adducts, as previously identified in Aβ*_16–22_-containing fibrils by Preston *et al*. (*16*), indicating that co-assembly into fibrils either is very rare and cannot be detected despite the sensitivity of ESI-MS or does not occur.

To provide a molecular image of co-assembly, we further analyzed the DMD simulations in which six Aβ_40_ and six Aβ_16–22_ monomers were mixed and their aggregation behavior was monitored versus time at *C*_peptide_ = 5 mM (Fig. 5D). The simulations showed that in the early stages of assembly (*t* = 0.6 μs), a mixture of monomeric and oligomeric Aβ_40_ was present. As the simulation progressed (*t* = 57 μs), all Aβ_40_ peptides coalesced into one β-sheet–rich oligomer, with Aβ_16–22_ intercalated within the structure. Throughout the simulation, monomeric Aβ_16–22_ was observed to bind transiently to other monomeric Aβ_16–22_ peptides or the KLVFF motif of Aβ_40_, in accordance with the data presented above. Last, at the end of the simulation (*t* = 202 μs), the peptides form distinct oligomeric domains, with Aβ_40_ and Aβ_16–22_ forming separate sheets.

### Aβ_40_ oligomer dynamics on the surface of Aβ_16–22_ fibrils

**Fig. 6:**
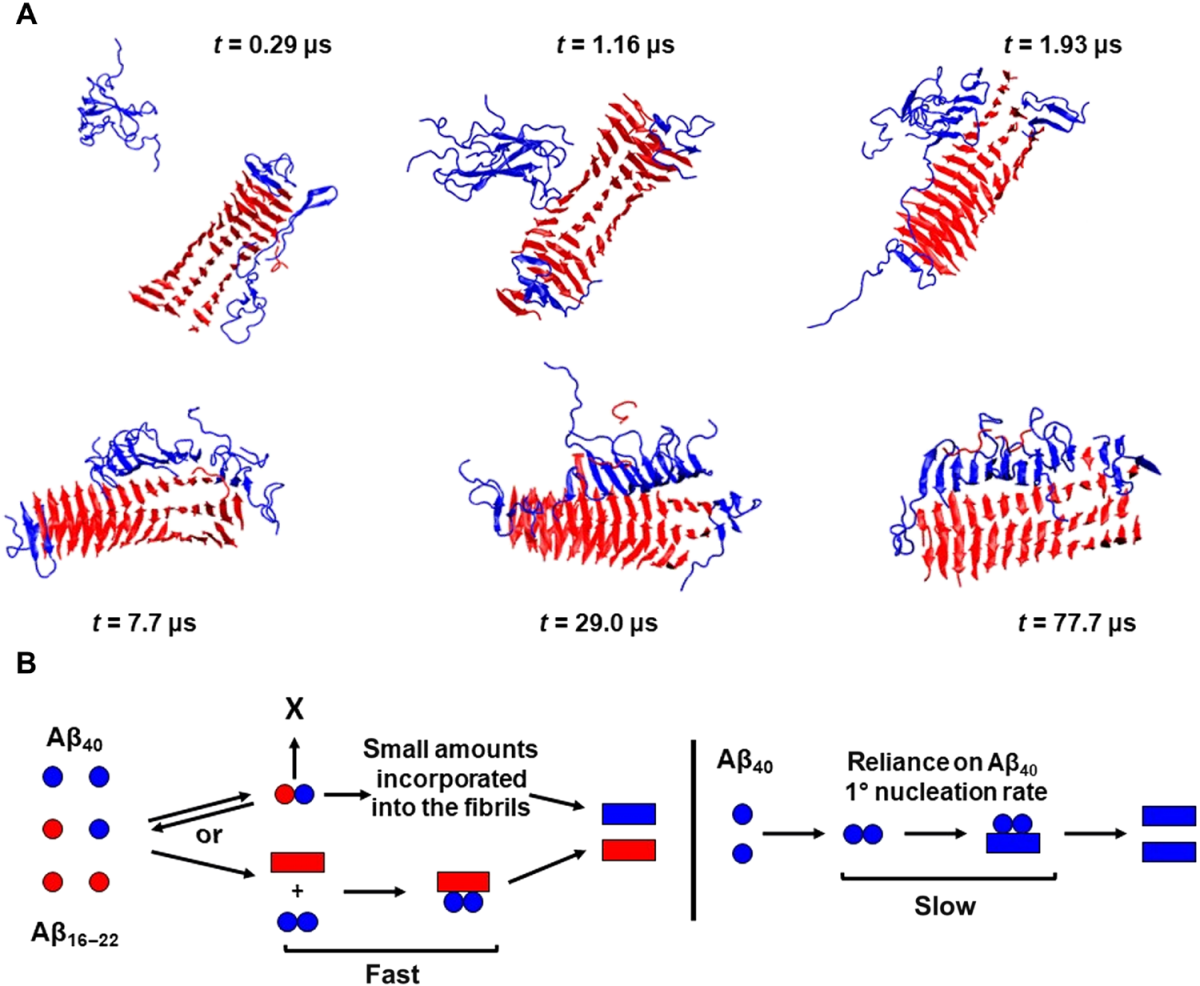
Aβ_16–22_fibrils catalyze Aβ_40_assembly through secondary surface nucleation. (**A**) Simulation snapshots of the process by which Aβ_16–22_ fibrils (red) increase the aggregation rate of Aβ_40_ (blue) through a surface-catalyzed secondary nucleation. (**B**) A schematic description of the mechanism is also included, with Aβ_40_ in blue and Aβ_16–22_ in red.

To obtain a molecular image of the process of secondary nucleation, we performed DMD simulations, in which six Aβ_40_ monomers were mixed with preformed fibrils of Aβ_16–22_ at *C*_Aβ40_ = 5 mM (Fig. 6A). At the early stage of the simulation (*t* = 0.29 μs), three Aβ_40_ peptides were present in an oligomer: One Aβ_40_ peptide was associated at the end of the fibril, and the remaining two Aβ_40_ peptides were elongated across the fibril surface. At this stage (*t* = 0.29 μs), the Aβ_40_ peptides in the oligomer and on the surface were observed to adopt a predominantly random coil conformation with small amounts of β-strand structure [note that an elongated monomeric structure was also observed in simulations performed by Barz and Strodel (*19*) in exploring the secondary nucleation of Aβ_42_ on the surface of Aβ_11–42_]. The β sheets were next observed to act as templates for peptides present in a random coil conformation (1.93 μs) and to pull them more fully to the fibril surface. Thus, as the simulation progressed, the Aβ_40_ peptides remaining in solution were recruited by those on the fibril surface. Once the oligomer became fully associated with the fibril surface, the amount of β-sheet structure in the surface-associated oligomer increased (*t* = 7.7 μs); antiparallel β strands formed via inter- and intramolecular hydrogen bonding, leading to sheet formation consistent with the early stages observed in the simulations performed for Aβ_40_ alone (*t* = 104 μs; Fig. 1C). Last, the surface-associated Aβ_40_ peptides were joined in an ordered oligomer (*t* = 29.0 and 77.7 μs). Related “bind and reorganize” processes for secondary nucleation were observed in simulations performed by Schwierz *et al.* (*38*) using Aβ_9–40_ as a model. As noted above, Aβ_40_ peptides attached both to the lateral surface and to the end of the Aβ_16–22_ fibril during the simulation, with the Aβ_40_ C-terminal region attaching more frequently to the lateral surface of the fibril than to the fibril ends at *C* = 5 mM (fig. S7). To assess the consistency of the results, we repeated the simulation three times; two of the three independent runs gave results similar to those described above, while for the final run, a greater number of associations to the fibril end were observed. Collectively, these results provide molecular images of surface-catalyzed nucleation, in which a random coil peptide is catalytically converted into a β-sheet fibrillar structure on a fibril surface.

## DISCUSSION

In this work, we used ESI-MS, PIC, and DMD to study the co-assembly mechanism of Aβ_16–22_ and Aβ_40_ into amyloid, demonstrating the power of using integrated approaches to study structural determinants of molecular assembly processes. We show that mixed Aβ_16–22_/Aβ_40_ heteromeric oligomers form but that these are transient and lowly populated (~1%) and do not significantly affect the rate of aggregation. In contrast, Aβ_16–22_ has a high propensity to self-associate into homomolecular fibrils, and these fibrils accelerate Aβ_40_ assembly by monomer/oligomer interactions through secondary nucleation at the fibril surface. Recent modeling of amyloid assembly kinetics has revealed the importance of primary nucleation, secondary nucleation, and fibril elongation in fibril growth mechanisms (*7*, *10*, *23*). Notably, a kinetic model has been described for the co-aggregation of Aβ_40/42_ (*24*). The experimental data presented here for co-aggregation of Aβ_16–22_ and Aβ_40_ qualitatively agree with this model, whereas our DMD simulations illustrate that while all primary/secondary nucleation and elongation processes occur simultaneously, secondary nucleation is the dominant process in Aβ_40_ fibril formation kinetics during co-assembly with Aβ_16–22_, which is consistent with the findings for the self-assembly mechanism of Aβ_40_ observed previously (*9*, *10*, *24*). Moreover, Aβ_40_ assembly intermediates on the surface of Aβ_16–22_ fibrils resemble those formed spontaneously in solution for Aβ_40_ alone, implying that the fibril surface catalyzes the assembly reaction without modifying the molecular mechanism, at least for the simulations performed here. Whether this holds for other sequences and co-assembly reactions will require further exploration (notably, which features of a fibril and the assembling monomer determine compatibility with secondary nucleation from a fibril surface).

Overall, the current study thus serves to emphasize the marked differences in aggregation behavior that are observed during co-aggregation compared to homomolecular self-assembly and underscores the need to use multiple methods to understand aggregation mechanisms in molecular detail. Significant current interest centers on characterizing distinct molecular steps leading to amyloid fibril formation, with secondary nucleation considered as playing a key role in causing toxicity (*11*, *39*). Recently, kinetic analyses have been augmented by mapping the free-energy landscapes defining different microscopic phases in the aggregation pathway (*11*), providing insight to facilitate development of strategies that modulate the thermodynamically distinct surface-monomer interactions characteristic of secondary nucleation. However, to design therapeutically useful modulators of amyloid aggregation requires that this understanding is complemented with structural insights of the molecular recognition between fibrils and monomers, set within the context of other interactions occurring during aggregation (e.g., monomer-nuclei interactions). We have shown here that Aβ_40_ monomers and oligomers dock onto the fibril surface, which catalyzes the assembly of antiparallel strand formation in close situ to the parent Aβ_16–22_ fiber. Whether this is the end-point product or further reorganization is required to generate the final amyloid structure requires further study (longer simulation time). In this context, metastable amyloid structures have been observed for the Iowa mutant of Aβ_40_ using solid-state NMR, in which antiparallel fibrils were observed as trapped intermediates in the assembly process to the final all-parallel fibril structure (*40*).

Together, the results demonstrate that kinetic analyses and theory together with MD provide a powerful arsenal and capability to visualize secondary nucleation in structural and kinetic detail. Such approaches may allow informed targeting of this process to either prevent or accelerate secondary nucleation for therapeutic purposes and peptide material assembly. Co-aggregation adds an additional layer of complexity in understanding molecular assembly yet represents an opportunity to manipulate these supramolecular assembly processes, as demonstrated here for the model system involving Aβ_16–22_ and Aβ_40_. Evidently, Aβ_40_ shows a propensity to aggregate via secondary nucleation from its own fibril surface or that of other peptide sequences, as shown here for fibrils of Aβ_16–22_. Hence, this work begins to address the molecular recognition events required for secondary nucleation to occur on a fibril surface and may inform strategies to modulate the aggregation of Aβ_40_ under conditions in which secondary nucleation dominates fibril growth.

## MATERIALS AND METHODS

### Synthesis of *N*-Fmoc TFMD-Phe and Aβ peptides

*N*-Fmoc TFMD-Phe was synthesized using the method described by Smith *et al.* (*41*) and further minor changes in protecting group (scheme S1). Aβ_16–22_, TAMRA-Ahx-Aβ_16–22_, and Aβ*_16–22_ were synthesized via both automated and manual solid-phase peptide synthesis and dissolved into DMSO stock solutions before use (fig. S1). Aβ_40_ was synthesized recombinantly using the method of Walsh *et al.* (*42*) and modifications by Stewart *et al.* (*43*). To ensure that Aβ_40_ was monomeric before use, the peptide was purified by size exclusion chromatography, lyophilized, and stored at −4°C (fig. S2).

### ThT fluorescence assays

Samples were prepared in a 96-well nonbinding plate (Corning Costar 3881, Corning Life Sciences, Amsterdam, the Netherlands) sealed with clear sealing film (BMG Labtech, Aylesbury, Bucks, UK) and were incubated in a FLUOstar OPTIMA plate reader (BMG Labtech, Aylesbury, Bucks, UK) for 20 hours at 37°C without agitation. Samples had a volume of 95 μl containing 10 μM ThT in 100 mM ammonium bicarbonate (pH 7.4) and a final concentration of 1% (v/v) DMSO. For seeding experiments, Aβ_16–22_ was incubated at 50 μM for at least 24 hours in the same buffer as described above, with the presence of fibrils confirmed by TEM (described below). Before the assay, the fibrils were probe-sonicated for 5 s at 22% amplitude to generate “seeds.” The ThT experiments used excitation and emission filters of 430 and 485 nm. Each ThT experiment shown was repeated in independent assays on three different occasions, with the traces shown in this work being representative of all repeats.

### Transmission electron microscopy

TEM images were taken at the end of each experiment by removing 5 μl from the necessary well and incubating this sample on carbon-formvar grids for 30 s before staining with 2% (w/v) uranyl acetate solution for an additional 30 s, as described by Preston *et al.* (*27*). Images were taken on a JEM-1400 (JEOL Ltd., Tokyo, Japan) or a Tecnai F12 TEM. Images were taken on a JEM-1400 (JEOL Ltd., Tokyo, Japan) or a Tecnai T12 (FEI, Hillsboro, OR, USA) TEM. Images were taken using either an ATM charge-coupled device (CCD) camera or a Gatan UltraScan 1000 XP (994) CCD camera (JEM-1400) or an UltraScan 100XP (994) CCD camera (Tecnai F12). Once taken, images were processed using ImageJ [National Institutes of Health (NIH)].

### General sedimentation protocol

Samples were taken at the desired time point and centrifuged (20 min, 14,000*g*, 4°C). Each sample was then separated into pellet and supernatant fractions, lyophilized overnight, and disaggregated in hexafluoroisopropanol (HFIP) for at least 2 hours. HFIP was removed under a stream of N_2_, and the peptides were taken up in DMSO before analysis by high-resolution MS (Bruker HCT ion-trap MS).

### Fluorescence quenching assays

Wild-type Aβ_16–22_ was spiked with 5% (w/w) TAMRA-Ahx-Aβ_16–22_ and incubated either in isolation or at a 1:1 ratio with Aβ_40_ (total peptide concentration, 40 μM) in 100 mM ammonium bicarbonate buffer (pH 7.4) with a final concentration of 2% (v/v) DMSO. Samples were placed in quartz cuvettes and analyzed using a temperature-controlled fluorimeter at 37°C. Time points were taken every 30 s for the duration of the experiment, and TEM images (as described above) were taken at the end of each experiment to ensure the presence of fibrils. The TAMRA fluorophore was excited at 520 nm, and emission was recorded at 600 nm to reduce the inner filter effect.

### ESI-IMS-MS analysis

All samples were prepared as described above and left to incubate at 37°C without agitation for 5 min. A SYNAPT HDMS quadrupole time-of-flight MS (Micromass UK Ltd., Waters Corp., Manchester, UK), equipped with a TriVersa NanoMate (Advion Biosciences, Ithaca, NY, USA) automated nano-ESI interface, was used in this study. The instrument has a traveling-wave IMS device situated in between the quadrupole and the time-of-flight analyzers, as described in detail elsewhere. Samples were analyzed by positive ionization nano-ESI, with a capillary voltage of 1.4 kV and a nitrogen-nebulizing gas pressure of 0.8 psi. The following instrumental parameters were set: cone voltage, 60 V; source temperature, 60°C; backing pressure, 4.7 mbar; ramped traveling speed, 7 to 20 V; traveling wave speed, 400 m s^−1^; IMS nitrogen gas flow, 20 ml min^−1^; IMS cell pressure, 0.55 mbar. The mass/charge ratio (*m*/*z*) scale was calibrated using aq. CsI cluster ions. CCS measurements were estimated using a calibration obtained by analysis of denatured proteins (cytochrome c, ubiquitin, and alcohol dehydrogenase) and peptides (tryptic digests of alcohol dehydrogenase and cytochrome c), with known CCSs obtained elsewhere from drift tube ion mobility measurements (*15*, *33*). Data were processed using MassLynx v4.1 and Driftscope software supplied with a mass spectrometer.

### Photo-induced covalent cross-linking

A 1:1 ratio of Aβ_16–22_/Aβ*_16–22_ or Aβ*_16–22_/Aβ_40_ (40 μM total peptide concentration) in 100 mM ammonium bicarbonate buffer (pH 7.4) with a final concentration of 1% (v/v) DMSO was incubated in Eppendorf tubes for either 5 min or 24 hours. Samples were then irradiated for 30 s using a homebuilt light-emitting diode lamp at 365 nm, then removed, lyophilized overnight, taken up in HFIP for at least 2 hours, and vortexed to ensure that any aggregates were disrupted. HFIP was then removed under a stream of N_2_, and the sample was resuspended in 50:50 (v/v) MeCN/H_2_O + 0.05% formic acid to a final concentration of ~40 μM. Any cross-links were then analyzed using the method previously described and the ESI-IMS-MS system as described above (*16*).

### DMD and PRIME20 force field

The simulation approach applied in this work is DMD, a fast alternative to traditional MD, in combination with the PRIME20 force field, a four-bead-per-residue coarse-grained protein model developed by the Hall group (*44*). In the PRIME20 model, each of the 20 different amino acids contains three backbone spheres (NH, C_α_H, and CO) and one side-chain sphere (R) with a distinct hard sphere diameter (effective van der Waals radius) and distinct side chain–to–backbone distances (R-CαH, R-NH, and R-CO). The backbone hydrogen bonding interaction is modeled as a directional square well potential. In the original PRIME20 force field, the potential function between any two side-chain beads on the 20 different amino acids (except glycine) is modeled as a single-well potential, containing 210 different square well widths and 19 different square well depths using the 5.5 Å heavy atom criteria. In this work, we follow Cheon’s approach to apply a double-square well potential instead of the single-square well for side chain–side chain interaction (*45*). All the other nonbonded interactions are modeled as hard sphere interactions. A detailed description of the derivation of the geometric and energetic parameters of the PRIME20 model is given in our earlier work (*46*).

### Simulation procedure

DMD/PRIME20 simulations were performed on the following systems: (i) six Aβ_40_ monomeric peptides; (ii) six monomeric Aβ_40_ peptides with six monomeric Aβ_16–22_ peptides; and (iii) six Aβ_40_ monomeric peptides in the presence of preformed two, three, and four β-sheet Aβ_16–22_ protofilaments. The two, three, and four β-sheet Aβ_16–22_ protofilaments contain 21, 42, and 71 peptides, respectively. Each simulation was performed at two different total peptide concentrations (1 and 5 mM). Similar seeding simulations were performed in a previous work (*45*). The simulations were performed in the canonical ensemble (fixed number of particles, volume, and temperature). The reduced temperature was defined to be T* = *k*_B_*T*/ε_HB_, where the hydrogen bonding energy ε_HB_ = 12.47 kJ/mol. The reduced temperature is related to real temperature by using the equation *T*/*K* = 2288.46T* − 115.79. The reduced temperature T* is chosen to be 0.20, which corresponds to a real temperature of 342 K. The system was maintained at a constant temperature by applying the Andersen thermostat. We performed 3 to 10 independent runs for each system.

## Supplementary Material

http://advances.sciencemag.org/cgi/content/full/5/6/eaav8216/DC1

Download PDF

Data file S1

Data file S2

Data file S3

Data file S4

Data file S5

Data file S6

Data file S7

Data file S8

Data file S9

Data file S10

## SUPPLEMENTARY MATERIALS

Supplementary material for this article is available at http://advances.sciencemag.org/cgi/content/full/5/6/eaav8216/DC1 General materials and methods for organic synthesis Synthesis of *N*-Fmoc–protected TFMD-Phe General materials and methods for Aβ_16–22_ solid-phase peptide synthesis General materials and methods for HPLC purification Analytical MS and HPLC data for synthetic peptides General materials and methods for recombinant peptide synthesis Additional characterization and analyses CCS analysis of Aβ_40_ in the presence and absence of Aβ_16–22_ Scheme S1. Synthesis of TFMD-Phe. Fig. S1. HRMS and analytical HPLC traces of Aβ_16–22_ and its variants. Fig. S2. SEC trace of Aβ_40_ indicates that there is a single peak, and ESI-IMS-MS indicates that in the gas phase Aβ_40_ is largely monomeric. Fig. S3. Supplementary ThT data. Fig. S4. Supplementary negative-stain TEM images. Fig. S5. Analysis of the CCS values for Aβ_40_ in the absence or presence of Aβ_16–22_ over different IMS experiments. Fig. S6. PIC analysis of 1:1 Aβ*_16–22_/Aβ_40_ at 5 min and 24 hours. Fig. S7. Plot of the average number of hydrogen bonding and side chain–side chain. Table S1. The expected and observed *m/z* values for monomeric and oligomeric Aβ_40_ in isolation and in the presence of a 1:1 ratio of Aβ_16–22_. Table S2. Assignments of each of the major peaks observed in fig. S6A. Data file S1. MD snapshots as pdb files Fig. 1 *t* = 0. Data file S2. MD snapshots as pdb files Fig. 1 *t* = 104. Data file S3. MD snapshots as pdb files Fig. 1 *t* = 230. Data file S4. MD snapshots as pdb files Fig. 1 *t* = 621. Data file S5. MD snapshots as pdb files Fig. 6 *t* = 0.29. Data file S6. MD snapshots as pdb files Fig. 6 *t* = 1.16. Data file S7. MD snapshots as pdb files Fig. 6 *t* = 1.93. Data file S8. MD snapshots as pdb files Fig. 6 *t* = 7.7. Data file S9. MD snapshots as pdb files Fig. 6 *t* = 29. Data file S10. MD snapshots as pdb files Fig. 6 *t* = 77.7.
