# Supplementary material for: Molecular insights into the surface-catalyzed secondary nucleation of amyloid-β40 (Aβ40) by the peptide fragment Aβ16–22
Source: Sci Adv. 2019 Jun 21;5(6):eaav8216. doi: 10.1126/sciadv.aav8216 (PMC6588359; doi:10.1126/sciadv.aav8216)
Supplement: http://advances.sciencemag.org/cgi/content/full/5/6/eaav8216/DC1 [file supp_5_6_eaav8216__index.html]

Science Advances | Science Advances

## Supplementary Materials

**The PDF file includes:**

- General materials and methods for organic synthesis
- Synthesis of *N*-Fmoc–protected TFMD-Phe
- General materials and methods for Aβ16–22 solid-phase peptide synthesis
- General materials and methods for HPLC purification
- Analytical MS and HPLC data for synthetic peptides
- General materials and methods for recombinant peptide synthesis
- Additional characterization and analyses
- CCS analysis of Aβ40 in the presence and absence of Aβ16–22
- Scheme S1. Synthesis of TFMD-Phe.
- Fig. S1. HRMS and analytical HPLC traces of Aβ16–22 and its variants.
- Fig. S2. SEC trace of Aβ40 indicates that there is a single peak, and ESI-IMS-MS indicates that in the gas phase Aβ40 is largely monomeric.
- Fig. S3. Supplementary ThT data.
- Fig. S4. Supplementary negative-stain TEM images.
- Fig. S5. Analysis of the CCS values for Aβ40 in the absence or presence of Aβ16–22 over different IMS experiments.
- Fig. S6. PIC analysis of 1:1 Aβ\*16–22/Aβ40 at 5 min and 24 hours.
- Fig. S7. Plot of the average number of hydrogen bonding and side chain–side chain.
- Table S1. The expected and observed *m/z* values for monomeric and oligomeric Aβ40 in isolation and in the presence of a 1:1 ratio of Aβ16–22.
- Table S2. Assignments of each of the major peaks observed in fig. S6A.

Download PDF

**Other Supplementary Material for this manuscript includes the following:**

- Data file S1 (.pdb format). MD snapshots as pdb files Fig. 1 *t* = 0.
- Data file S2 (.pdb format). MD snapshots as pdb files Fig. 1 *t* = 104.
- Data file S3 (.pdb format). MD snapshots as pdb files Fig. 1 *t* = 230.
- Data file S4 (.pdb format). MD snapshots as pdb files Fig. 1 *t* = 621.
- Data file S5 (.pdb format). MD snapshots as pdb files Fig. 6 *t* = 0.29.
- Data file S6 (.pdb format). MD snapshots as pdb files Fig. 6 *t* = 1.16.
- Data file S7 (.pdb format). MD snapshots as pdb files Fig. 6 *t* = 1.93.
- Data file S8 (.pdb format). MD snapshots as pdb files Fig. 6 *t* = 7.7.
- Data file S9 (.pdb format). MD snapshots as pdb files Fig. 6 *t* = 29.
- Data file S10 (.pdb format). MD snapshots as pdb files Fig. 6 *t* = 77.7.

**Files in this Data Supplement:**

- Adobe PDF - aav8216\_SM.pdf
