# Supplementary material for: Molecular insights into the surface-catalyzed secondary nucleation of amyloid-β40 (Aβ40) by the peptide fragment Aβ16–22
Source: Sci Adv. 2019 Jun 21;5(6):eaav8216. doi: 10.1126/sciadv.aav8216 (PMC6588359; doi:10.1126/sciadv.aav8216)
Supplement: Download PDF [file aav8216_SM.pdf]

Science Advances
AAAS

advances.sciencemag.org/cgi/content/full/5/6/eaav8216/DC1

Supplementary Materials for

Molecular insights into the surface-catalyzed secondary nucleation of amyloid-β_{40} (Aβ_{40}) by the peptide fragment A$\beta_{16–22}$

Samuel J. Bunce, Yiming Wang, Katie L. Stewart, Alison E. Ashcroft, Sheena E. Radford*, Carol K. Hall*, Andrew J. Wilson*

*Corresponding author. Email: s.e.radford@leeds.ac.uk (S.E.R.); hall@ncsu.edu (C.K.H.); a.j.wilson@leeds.ac.uk (A.J.W.)

Published 21 June 2019, *Sci. Adv.* **5**, eaav8216 (2019)
DOI: 10.1126/sciadv.aav8216

The PDF file includes:

General materials and methods for organic synthesis
Synthesis of *N*-Fmoc–protected TFMD-Phe
General materials and methods for A$\beta_{16–22}$ solid-phase peptide synthesis
General materials and methods for HPLC purification
Analytical MS and HPLC data for synthetic peptides
General materials and methods for recombinant peptide synthesis
Additional characterization and analyses
CCS analysis of Aβ_{40} in the presence and absence of A$\beta_{16–22}$
Scheme S1. Synthesis of TFMD-Phe.
Fig. S1. HRMS and analytical HPLC traces of A$\beta_{16–22}$ and its variants.
Fig. S2. SEC trace of Aβ_{40} indicates that there is a single peak, and ESI-IMS-MS indicates that in the gas phase Aβ_{40} is largely monomeric.
Fig. S3. Supplementary ThT data.
Fig. S4. Supplementary negative-stain TEM images.
Fig. S5. Analysis of the CCS values for Aβ_{40} in the absence or presence of A$\beta_{16–22}$ over different IMS experiments.
Fig. S6. PIC analysis of 1:1 A$\beta^*_{16–22}$/Aβ_{40} at 5 min and 24 hours.
Fig. S7. Plot of the average number of hydrogen bonding and side chain–side chain.
Table S1. The expected and observed *m/z* values for monomeric and oligomeric Aβ_{40} in isolation and in the presence of a 1:1 ratio of A$\beta_{16–22}$.
Table S2. Assignments of each of the major peaks observed in fig. S6A.

Other Supplementary Material for this manuscript includes the following:

(available at advances.sciencemag.org/cgi/content/full/5/6/eaav8216/DC1)

Data file S1 (.pdb format). MD snapshots as pdb files Fig. 1 t = 0.
Data file S2 (.pdb format). MD snapshots as pdb files Fig. 1 t = 104.
Data file S3 (.pdb format). MD snapshots as pdb files Fig. 1 t = 230.
Data file S4 (.pdb format). MD snapshots as pdb files Fig. 1 t = 621.
Data file S5 (.pdb format). MD snapshots as pdb files Fig. 6 t = 0.29.
Data file S6 (.pdb format). MD snapshots as pdb files Fig. 6 t = 1.16.
Data file S7 (.pdb format). MD snapshots as pdb files Fig. 6 t = 1.93.
Data file S8 (.pdb format). MD snapshots as pdb files Fig. 6 t = 7.7.
Data file S9 (.pdb format). MD snapshots as pdb files Fig. 6 t = 29.
Data file S10 (.pdb format). MD snapshots as pdb files Fig. 6 t = 77.7.

Supplementary Materials

General materials and methods for organic synthesis

Non-aqueous reactions were carried out in washed and oven-dried glassware. Solvents and reagents were used as received from major suppliers without prior purification unless stated. Anhydrous tetrahydrofuran (THF), ethanol (EtOH), acetonitrile (MeCN), dichloromethane (DCM) and diethyl ether (Et_2O) were obtained from the in-house solvent purification system from Innovative Technology Inc. PureSolv®. Anhydrous dimethyl formamide (DMF), methanol (MeOH) and chloroform ($CHCl_3$) were obtained from major chemical suppliers equipped with a SureSeal™ (or equivalent). For reactions under non-anhydrous conditions, the solvents used were of HPLC quality and provided by Fisher or Sigma-Aldrich. Water in aqueous solutions and used for quenching was deionized, and water used for buffers and HPLC was ultra-pure 18 MΩ from an ELGA Purelab system. ^{1}H NMR spectra were recorded on Bruker DPX 300 (300 MHz) or Avance 500 (500 MHz) spectrometers and referenced to either residual non-deuterated solvent peaks or tetramethylsilane. ^{1}H spectra are reported as follows: δ_H (spectrometer frequency, solvent): ppm to two decimal places (number of protons, multiplicity, *J* coupling constant in hertz, assignment). Chemical shifts are quoted in ppm with signal splitting recorded as singlet (s), doublet (d), triplet (t), quartet (q), quintet (quin.) multiplet (m), broad (br) and apparent (app.). Coupling constants, *J*, are measured to the nearest 0.1 Hz.

Synthesis of *N*-Fmoc–protected TFMD-Phe

The synthesis of TFMD-Phe was carried out according to the literature procedure originally developed by Fishwick and co-workers and improved upon by Smith and co-workers.[5,6] A change in protecting group has been made from the original Smith and co-workers synthesis. All synthetic products have been analysed by at least ^{1}H NMR to confirm the identity and purity of each compound. Principally, ^{1}H NMR, FTIR and LC-MS analysis were used. All data has been consistent with those previously reported within the group and in the literature.[6,7]

Scheme S1. Synthesis of TFMD-Phe.

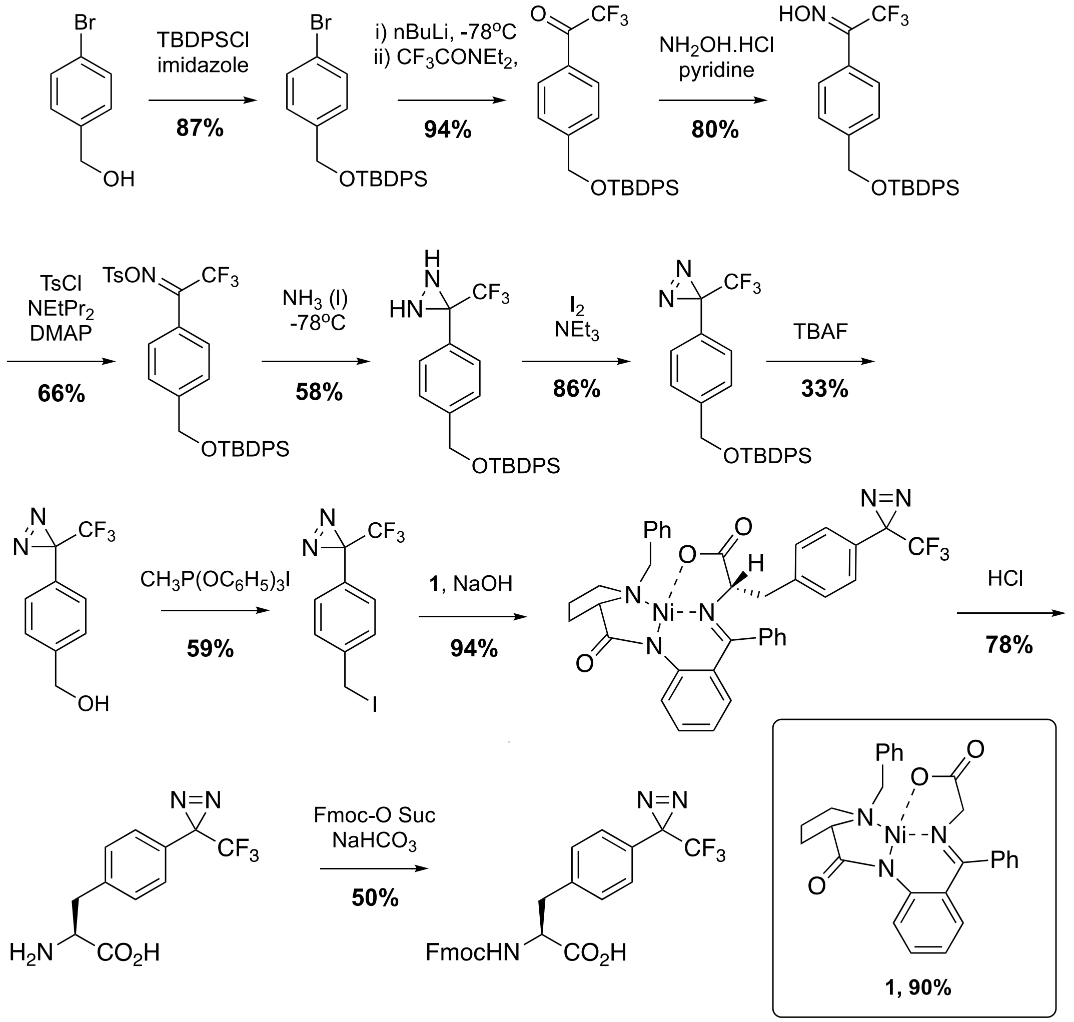

General materials and methods for A$\beta_{16–22}$ solid-phase peptide synthesis

All amino acids, coupling reagents and resins were purchased from Novasyn (Merck), Fluorochem or Sigma-Aldrich. All amino acids were *N*-Fmoc protected and side chains were protected with Boc (Lys) or OtBu (Glu). Aβ_{16-22}, TAMRA-Ahx-Aβ_{16-22} and Aβ^*_{16-22} were synthesized using standard SPPS conditions on an automated solid-phase peptide synthesiser (CEM LibertyBlue) for Aβ_{16-22} and TAMRA-Ahx-Aβ_{16-22} or manually for Aβ^*_{16-22}. DMF, DCM and Et_2O used in peptide synthesis was of ACS grade from Sigma-Aldrich. Two different N-termini capping strategies were used:

N-terminal acetyl capping (Aβ_{16-22} and Aβ^*_{16-22}): The deprotected resin was washed with DMF (5 x 2 min x 2 mL), DCM (5 x 2 min x 2 mL) and Et_2O (5 x 2 min x 2 mL). After this acetic anhydride (10 equivalents) and DIPEA (10 equivalents) were added to the resin, which was agitated overnight on a blood spinner at room temperature.
N-terminal TAMRA labelling (TAMRA-Ahx-Aβ_{16-22}): Prior to coupling of the fluorescent label a 6-aminohexanoic acid (6-Ahx) linker would be coupled using standard SPPS conditions. For fluorescent labelling, TAMRA (5 equivalents) was dissolved in a solution of DMF (2 mL) containing oxyma (5 equivalents), DIC (5 equivalents) and left to stir for 10 mins. The pre-activated TAMRA solution was added to resin, which was agitated overnight on a blood spinner at room temperature in the dark.

After cleavage from the resin using TFA:TIPS:H_2O (98:1:1, 3 mL, 2 hr) the crude product was precipitated using ice cold Et_2O (50 mL) and the supernatant removed. The crude peptides identity was confirmed by LC-MS prior to HPLC purification.

General materials and methods for HPLC purification

Peptides were purified by preparative scale HPLC using an X-bridge C18 preparative column (reversed phase) on an increasing gradient of MeCN to H_2O. Crude peptides were dissolved in DMSO and injected into the column in 300 µL aquilots. The solvent gradient (5 – 95% MeCN in H_2O + 0.1% formic acid) was increased linearly over a 15 min run time at a flow rate of 10 mL/min^{-1}. The fixed-wavelength detector was set to scan the eluent at either 220, 254 or 270 nm with peak-based collection for 30 s after the diode was triggered. Fractions were analyzed by LC-MS and fractions containing the desired peptide were pooled, concentrated under reduced pressure and lyophilized. The purity of the peptide samples was determined by the School of Chemistry HPLC service. Identity was confirmed by mass using either the LC-MS or HRMS.

Analytical MS and HPLC data for synthetic peptides

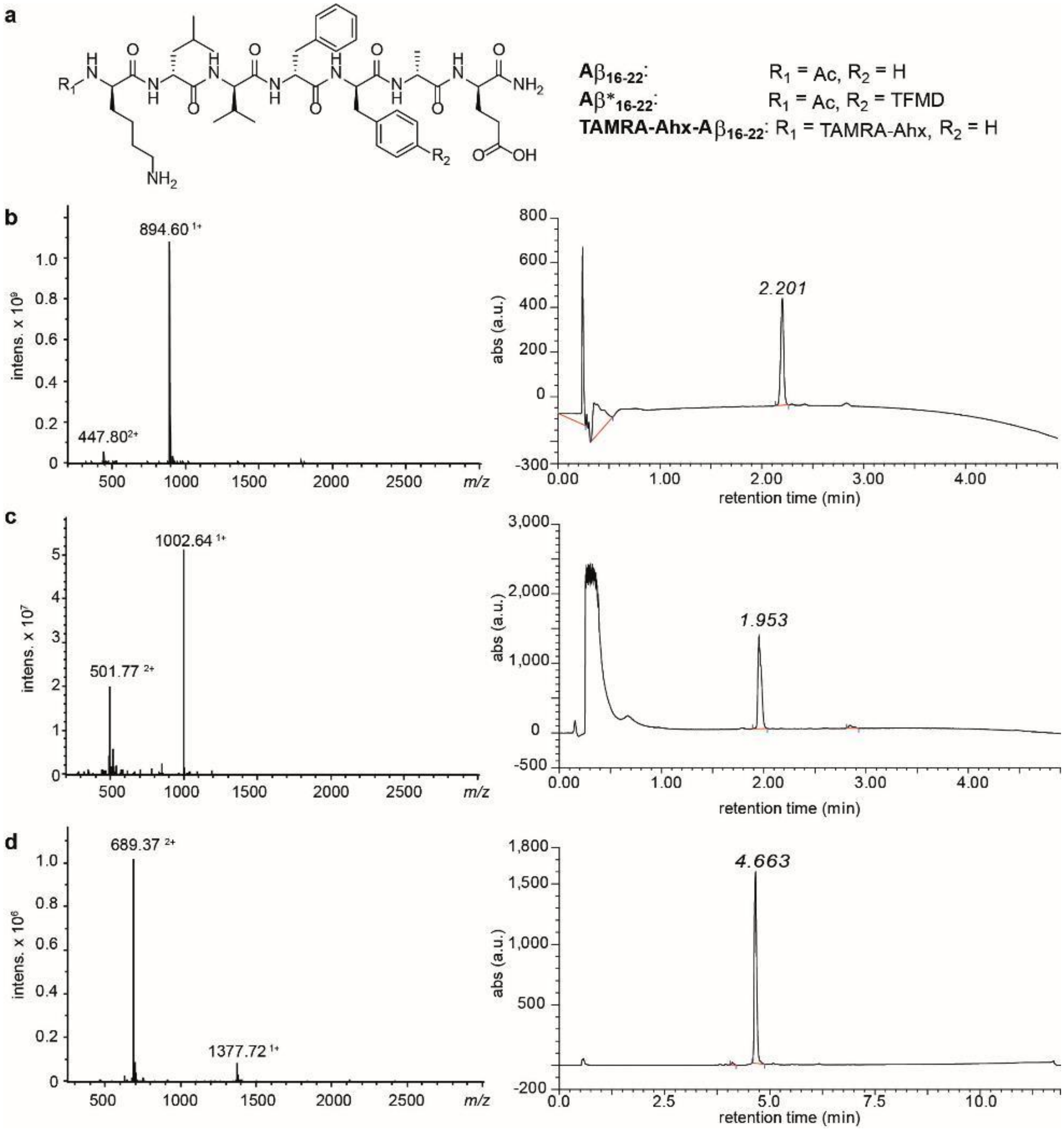

Fig. S1. HRMS and analytical HPLC traces of A$\beta_{16–22}$ and its variants. The core molecular structure of Aβ_{16-22} (a) and the associated traces for Aβ_{16-22} (b), Aβ^*_{16-22} (c) and TAMRA-Ahx-Aβ_{16-22} (d) are shown.

General materials and methods for recombinant peptide synthesis

Commercial *E. coli* strain BL21 (DE3) cells (Agilent) were transformed with a pETSAC plasmid containing the sequences for Aβ_{40} (a gift from Profs. Sara Linse and Dominic Walsh), including an N-terminal methionine residue that has no effect on the fibrillation of Aβ_{40} or the morphology of fibrils formed.(*44*) Cultures were grown in LB media and Aβ_{40} purified as described previously.(*45*) Final protein concentrations were estimated from UV absorption in 6 M guanidinium hydrochloride at 280 nm using an extinction coefficient of 1490 $M^{-1}cm^{-1}$, and averaged 4 mg pure peptide/L culture. HRMS was used to confirm the identity of the final product (expected molecular weight 4459.21 Da). The size exclusion chromatography trace from the second purification step (in 50 mM ammonium bicarbonate) is also shown. Fraction 11 and 12 were pooled and lyophilised prior to use.

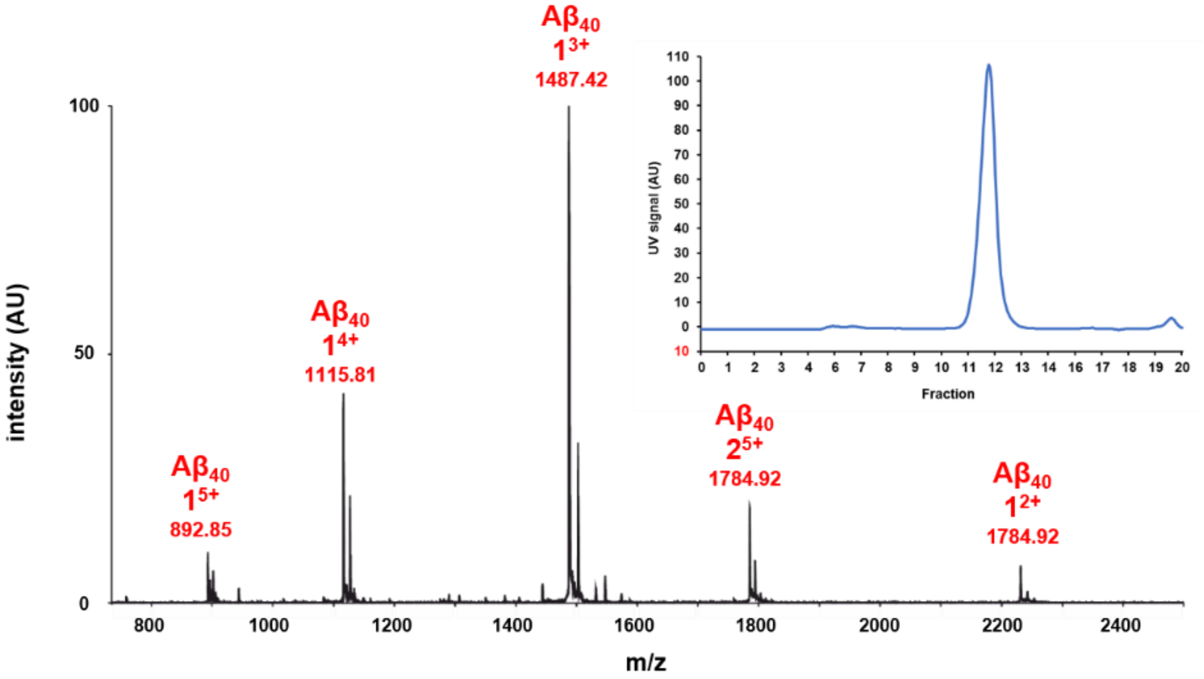

Fig. S2. SEC trace of Aβ_{40} indicates that there is a single peak, and ESI-IMS-MS indicates that in the gas phase Aβ_{40} is largely monomeric. The observed *m/z* ratio is stated above each peak.

Additional Characterization and Analyses

a

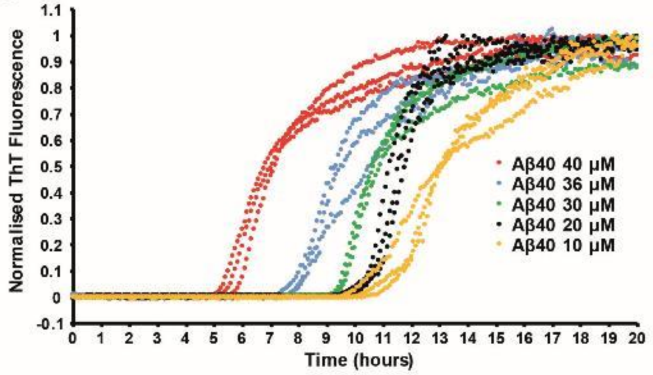

$A\beta_{40}$ concentration (μM)	Half-time (hours)
40	6.99± 0.14
36	9.74± 0.58
30	10.72± 0.18
20	11.53± 0.21
10	13.27± 0.46

b

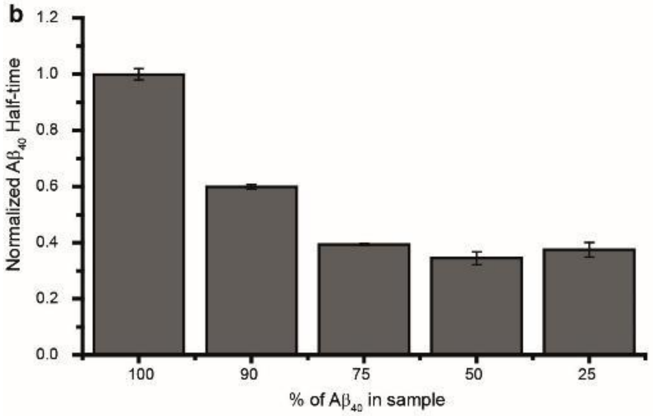

$A\beta_{40}$ in sample (%)	Normalized reduction in half-time
100	99.9± 4.6
90	59.3± 0.5
75	39.1± 0.4
50	33.3± 2.5
25	37.5± 2.6

c

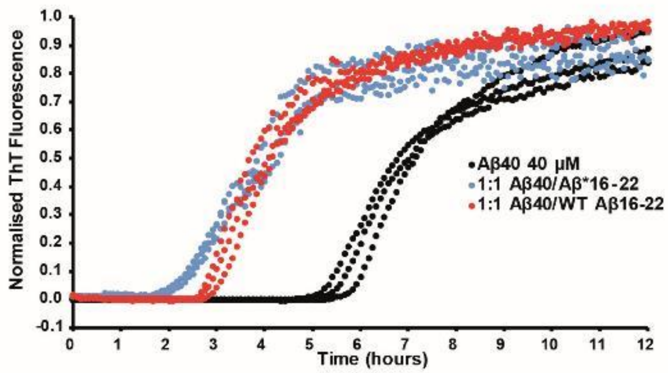

d

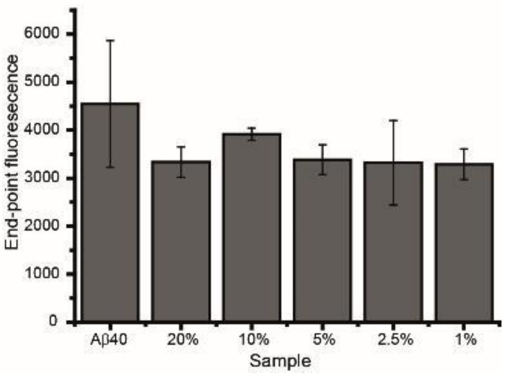

Fig. S3. Supplementary ThT data. The $A\beta_{40}$ concentration series (a) used to normalise the half-times of $A\beta_{40}$ aggregation in the presence of $A\beta_{16-22}$ (see main text) (b). $A\beta*_{16-22}$ (blue) has the same effect on the aggregation rate of $A\beta_{40}$ as WT $A\beta_{16-22}$ (red) (c). The end point ThT fluorescence data when increasing amounts of preformed $A\beta_{16-22}$ fibrils are added to $A\beta_{40}$ show no significant difference in fluorescence, indicating that homomolecular fibrils are formed (at least predominantly) at the end of the self-assembly reaction (d). Conditions: 100 mM ammonium bicarbonate, pH 7.4 with a final total peptide concentration of 40 μM in 1% (v/v) DMSO, 37 °C, quiescent.

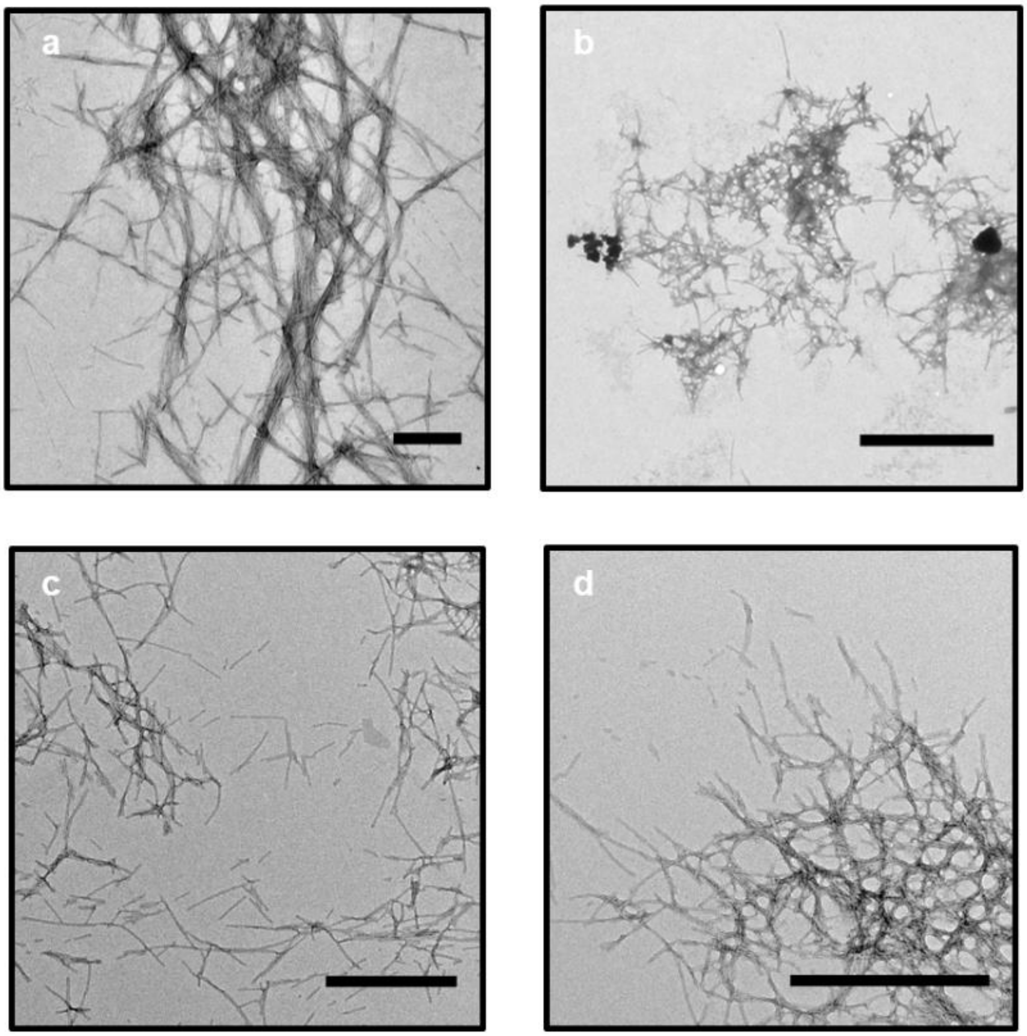

Fig. S4. Supplementary negative-stain TEM images. A$\beta_{16\text{-}22}$ forms fibrils that bundle together (a), after probe sonication the fibrils are shorter and have more fibril ends per aggregate mass (b). Conditions: total peptide concentration 50 µM, 100 mM ammonium bicarbonate, pH 7.4 with a final concentration of 1% (v/v) DMSO, sonication 5 s at 22% amplitude. When incubated at 5% (w/w) of the total peptide concentration, mixtures of TAMRA-Ahx-A$\beta_{16\text{-}22}$ and WT A$\beta_{16\text{-}22}$ form fibrils that are morphologically similar to those formed by WT A$\beta_{16\text{-}22}$ (c and d (zoomed)). Conditions: total peptide concentration 40 µM, 100 mM ammonium bicarbonate, pH 7.4 with a final concentration of 2% (v/v) DMSO, quiescent, 37 °C. Scale bar = 500 nm.

Table S1. The expected and observed *m/z* values for monomeric and oligomeric Aβ_{40} in isolation and in the presence of a 1:1 ratio of A$\beta_{16\text{–}22}$. The expected and observed *m/z* of the heteromeric oligomers of Aβ_{40}/A$\beta_{16\text{-}22}$ are also shown (bottom).

Monomers

Peak	Aβ_{40} alone m/z expected	Aβ_{40} alone m/z observed	1:1 Aβ_{40}/A$\beta_{16\text{-}22}$ m/z expected	1:1 Aβ_{40}/A$\beta_{16\text{-}22}$ m/z observed
1^{2+}	2230.60	2230.79	2230.60	2230.79
1^{3+}	1487.40	1487.50	1487.40	1487.50
1^{4+}	1115.80	1115.88	1115.80	1115.88
1^{5+}	892.84	892.91	892.84	892.91
A$\beta_{16\text{-}22}$ 1^{1+}	N/A	N/A	894.49	894.58

Dimers

Peak	Aβ_{40} alone m/z expected	Aβ_{40} alone m/z observed	1:1 Aβ_{40}/A$\beta_{16\text{-}22}$ m/z expected	1:1 Aβ_{40}/A$\beta_{16\text{-}22}$ m/z observed
2^{4+}	2230.60	2230.79	2230.60	2230.79
2^{5+}	1784.68	1784.98	1784.68	1784.98
2^{6+}	1487.40	1487.42	N/A	N/A

Trimers, Tetramers and Pentamers

Peak	Aβ_{40} alone m/z expected	Aβ_{40} alone m/z observed	1:1 Aβ_{40}/A$\beta_{16\text{-}22}$ m/z expected	1:1 Aβ_{40}/A$\beta_{16\text{-}22}$ m/z observed
3^{5+}	2676.53	2677.14	2676.53	2677.14
3^{6+}	2230.60	2230.79	N/A	N/A
4^{6+}	2973.81	2974.03	N/A	N/A
4^{7+}	2549.12	2549.81	N/A	N/A
5^{8+}	2788.00	2788.9	N/A	N/A

Heteromeric species (Aβ_{40}/ A$\beta_{16\text{-}22}$)

Peak	m/z expected	m/z observed
$1:1^{3+}$	1785.33	1785.37
$1:2^{3+}$	2083.17	2083.58
$1:3^{3+}$	2381.00	2181.73
$2:1^{5+}$	1963.50	1963.95
$2:2^{5+}$	2142.20	2142.71
$2:3^{5+}$	2320.90	2321.19

Collision Cross-section (CCS) analysis of Aβ$_{40}$ in the presence and absence of Aβ$_{16–22}$

Collision cross-section (CCS) measurements were estimated by use of a IMS-MS calibration obtained by analysis of denatured proteins (cytochrome c, ubiquitin, alcohol dehydrogenase) and peptides (tryptic digests of alcohol dehydrogenase and cytochrome c) with known CCSs obtained elsewhere from drift tube ion mobility measurements.[3,4] The proteins were denatured in 50/:50 H_2O/MeCN + 0.1% (v/v) formic acid prior to introduction to the mass spectrometer. The CCS (Ω) of the peptide monomers/oligomers was then calculated according to the equation below

$$\Omega\,(\text{Å}^2) = A \times (t_D)^B \times z \times \sqrt{\frac{1}{m_{ion}} + \frac{1}{m_{gas}}}$$

where A is the determined calibration constant, z is the charge state of the ion, B is the exponential factor (determined experimentally), $t_D{}'$ is the corrected absolute drift time, m_{ion} is the mass of the ion and m_{gas} is the mass of the gas used in the ion-mobility cell (N_2).

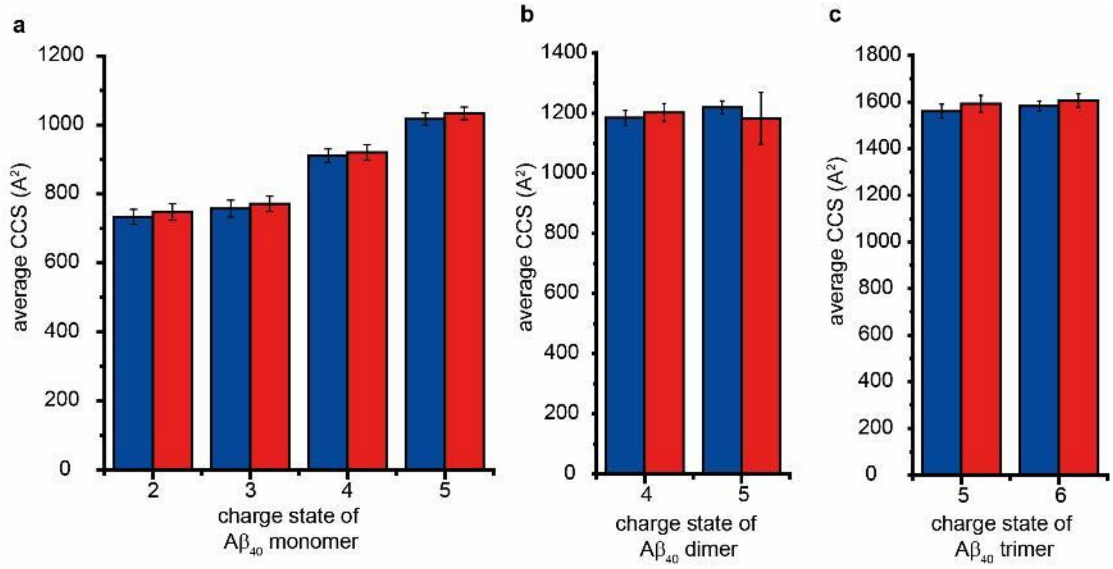

Fig. S5. Analysis of the CCS values for Aβ$_{40}$ in the absence or presence of Aβ$_{16–22}$ over different IMS experiments. The data are separated into monomer (a), dimer (b) and trimer (c) and broken down by charge state. The error bars represent the standard deviation over four measurements.

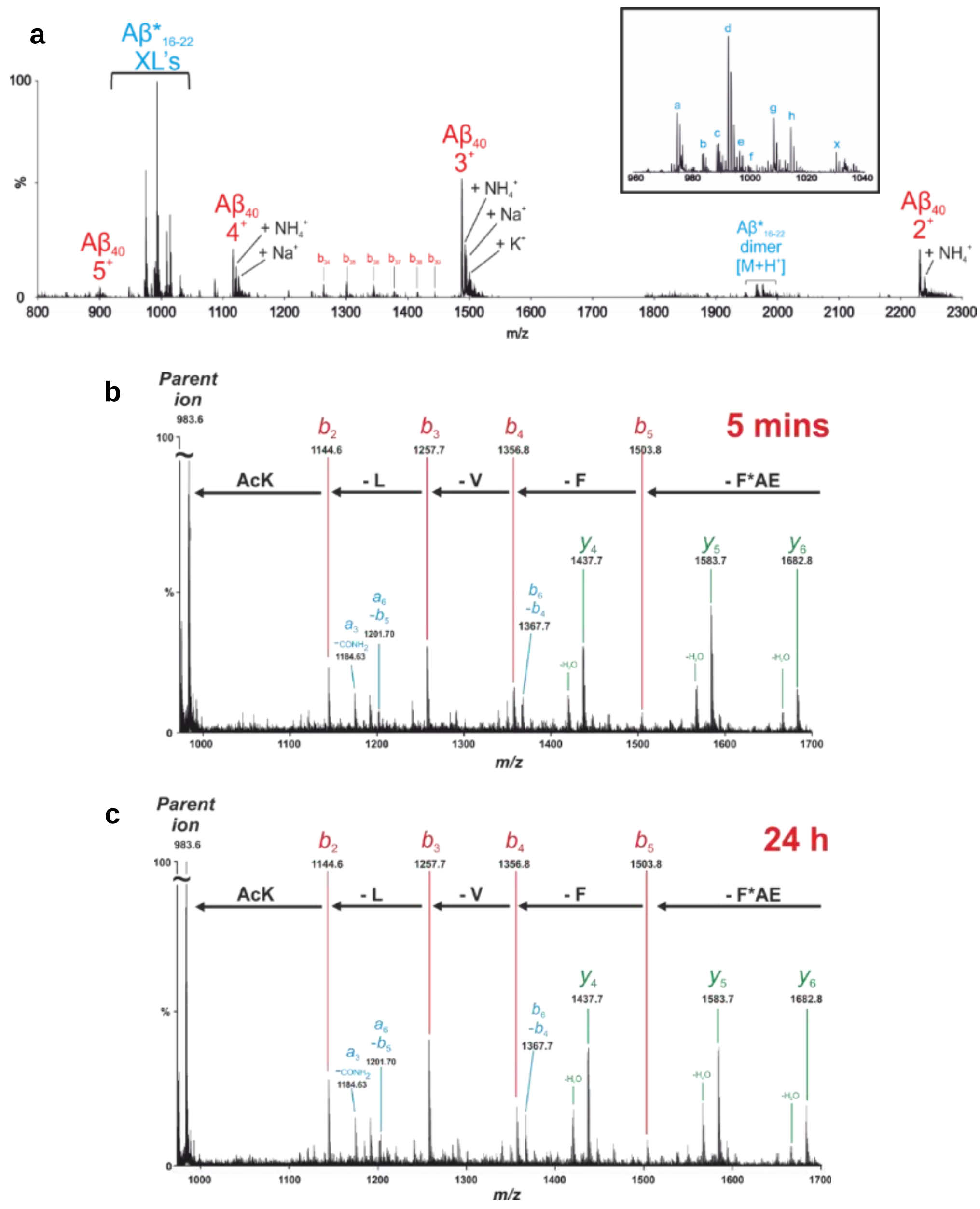

Fig. S6. PIC analysis of 1:1 Aβ*$_{16–22}$/Aβ$_{40}$ at 5 min and 24 hours. A full scan of the cross-linked region of m/z 800 – 2300 is shown at 5 mins (with the Aβ*$_{16-22}$ cross-linked region shown as the inset, the assignments can be found in Table S2 (a). Tandem MS/MS spectra of one of the dimer peaks (m/z 893.5) is shown at 5 mins (b) and 24 h (c), demonstrating that Aβ*$_{16-22}$ forms in-register antiparallel fibrils.

Table S2. Assignments of each of the major peaks observed in fig. S6A.

Peak	Charge	*m/z*	Structure	Comments	Observed at 24 h?
a	1+	974.5	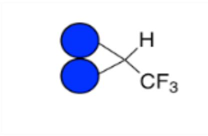	intramolecular cross-link (e = a + Na^+)	Yes
b	2+	983.5	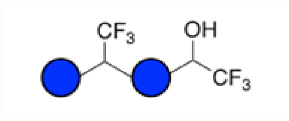	intermolecular Aβ*$_{16\text{-}22}$ - Aβ*$_{16\text{-}22}$ cross-link	Yes
c	2+	988.5	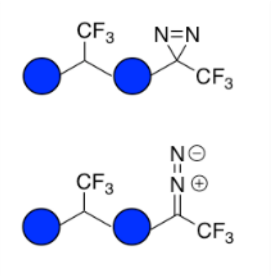	intermolecular Aβ*$_{16\text{-}22}$ - Aβ*$_{16\text{-}22}$ cross-link	Yes
d	1+	992.6	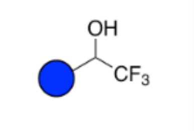	H_2O adduct (g = d + Na^+)	Yes
f	1+	1002.5	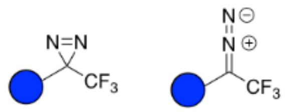	Parent ion/diazoisomer	No
h	1+	1008.5	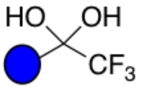	double H_2O adduct	Yes

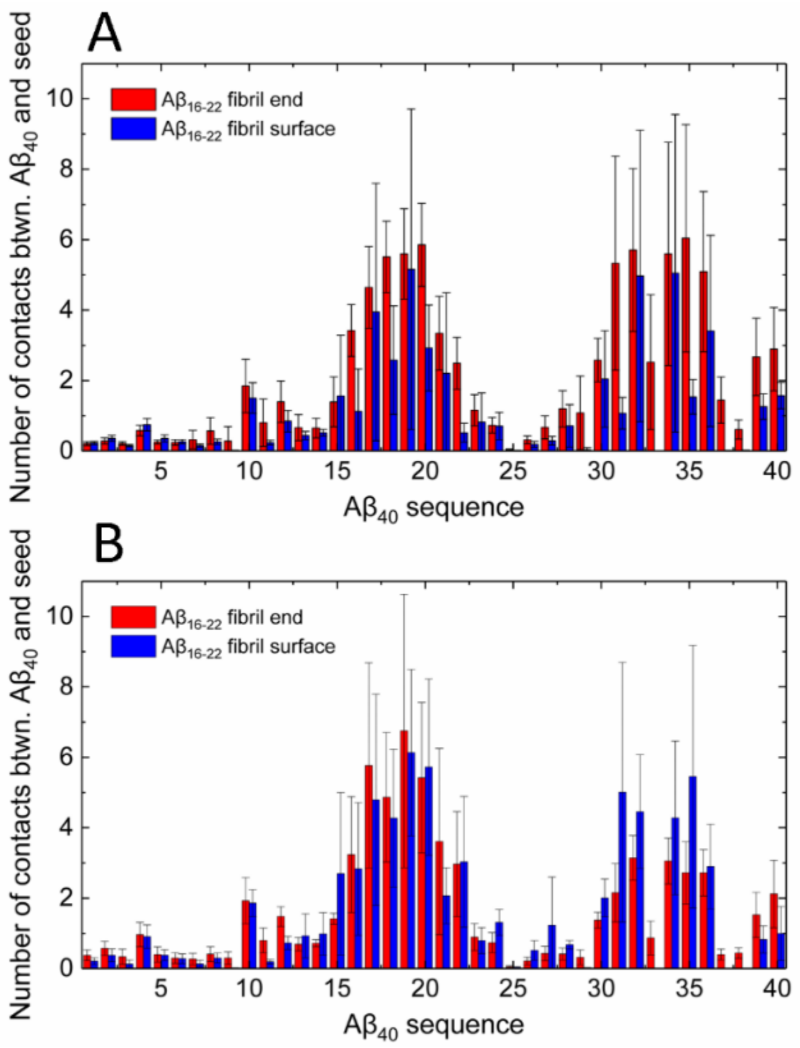

Fig. S7. Plot of the average number of hydrogen bonding and side chain–side chain. contacts between six Aβ_{40} monomers and a preformed 3-β-sheet A$\beta_{16\text{-}22}$ fibril during the simulation at C Aβ_{40} = 1mM (**A**) and 5 mM (**B**). As shown in Fig. 6, Aβ_{40} monomers attach to both the lateral surface and the end of the A$\beta_{16\text{-}22}$ fibril. Error bars depict the standard deviation calculated from three independent runs of the seeding simulation.

PDB Files for individual snapshots are included as Supporting Data files number according to the figure and time at which they appear e.g. Fig 1c-0.pdb, Fig 1c-104.pdb.
